# Mental health treatment for people with dual diagnosis

**DOI:** 10.1192/j.eurpsy.2025.250

**Published:** 2025-08-26

**Authors:** L. Lien

**Affiliations:** Health and welfare, University of Inlandet, Elverum, Norway

## Abstract

**Abstract:**

In this presentation I will show that mental health treatment for people with dual diagnosis is not very different from treatment of mental disorders without addiction problems. There are a few points that will be raised like interactions with the drug of choice for the patients and the problem arising from compliance.

The most important point that will be presented is the fact that too many people with dual disorders go untreated. There is a need to rise awareness of this problem within our profession.

The main aim of treatment is often to replace some of the effects that the patients experience using drugs like depression, irritability, fluctuating thoughts and sleep problems.

**Disclosure of Interest:**

None Declared

